# Correlations between clinical features and MRI findings in early adhesive capsulitis of the shoulder: a retrospective observational study

**DOI:** 10.1186/s12891-020-03569-8

**Published:** 2020-08-13

**Authors:** Yoon-Hee Choi, Dong Hyun Kim

**Affiliations:** 1 Department of Physical Medicine and Rehabilitation, Soonchunhyang University Hospital Seoul, Soonchunhyang University College of Medicine, Seoul, 04401 South Korea; 2 Department of Radiology, Seoul Metropolitan Government - Seoul National University Boramae Medical Center, Seoul National University College of Medicine, Seoul, 07061 South Korea

**Keywords:** Shoulder, Adhesive capsulitis, Magnetic resonance imaging, Range of motion, pain

## Abstract

**Background:**

This retrospective study investigated the association between clinical features and MRI findings in patients with early adhesive capsulitis of the shoulder.

**Methods:**

The study included 29 patients with early adhesive capsulitis of the shoulder. The clinical diagnostic criteria were significantly restricted passive range of motion (ROM) and a symptom duration of up to 9 months. Various measurements related to adhesive capsulitis, including humeral and glenoid capsular thickness in the axillary recess, maximal axillary capsular thickness, coracohumeral ligament thickness, and anterior capsular thickness were measured on MRI. Abnormal humeral and glenoid capsular hyperintensity in the axillary recess, abnormal hyperintensity in the rotator interval, and obliteration of the subcoracoid fat triangle were also evaluated. Correlations between MRI findings and clinical features, including limited ROM, pain, and symptom duration were sought.

**Results:**

Maximal axillary and humeral capsular thickness measured on MRI were negatively correlated with ROM for internal rotation. Also, hyperintensity in axillary recess and glenoid capule were correlated with ROM for abduction. Humeral capsular hyperintensity was correlated with ROM for forward flexion. There were no MRI findings that showed correlations with ROM for external rotation and severity of pain. The hyperintensity in the humeral capsule among MRI findings was only correlated with duration of symptoms.

**Conclusions:**

MRI can be useful for assessment of several measures of clinical impairment in patients with adhesive capsulitis. Thickening and hyperintensity of the joint capsule in the axillary recess on MRI is associated with limited ROM and duration of symptoms.

## Background

Adhesive capsulitis (AC) of the shoulder is characterized by shoulder pain and limited active and passive range of motion (ROM) in the shoulder [1–3]. In the past, the diagnostic terminology for this entity, such as “frozen shoulder,” was ambiguous and based on clinical features and symptoms [3, 4]. However, the disease presents with characteristic pathophysiological features, including capsular thickening and fibrosis due to chronic inflammation of the joint capsule, which may lead to capsular adhesion [1, 5].

Given that other diseases of the shoulder, such as rotator cuff tear, bursitis, and calcified tendinitis, may have similar clinical symptoms [1, 6], magnetic resonance imaging (MRI), ultrasound, and magnetic resonance (MR) arthrography are useful for differentiating AC from other diseases [2, 7–10]. MRI offers better resolution and soft tissue contrast than other methods and is a key modality for differentiating shoulder disease [2]. Based on previous studies that used MRI, the key diagnostic findings for AC include capsular thickening, a hyperintense T2 signal and contrast enhancement in the axillary capsule and rotator interval, thickening of the coracohumeral ligament (CHL), and obliteration of the subcoracoid fat triangle [2, 11, 12]. These MRI findings have an important role in the diagnosis of early AC when clinical features are atypical and in shortening the duration of joint stiffness by allowing timely physical therapy and intra-articular steroid injection, which could help to reduce the morbidity rate [13, 14]. Although diverse structural abnormalities are known to be associated with AC, there is limited literature on the association between radiologic findings and clinical features, and the few studies available have assessed several MRI findings with some ROMs (external rotation and abduction) [9], or some MRI findings with several ROMs [15–17]. Particularly it is known that, in pathophysiology, the capsule thickening and hypervascularization gradually progress in the early stages of AC disease [9, 15]. We hypothesized that these changes in AC were measured by MRI and could be related to the severity of clinical findings such as the patient’s ROM and shoulder pain. Accordingly, the objective of the present study was to investigate the association between various clinical features and the MRI findings for early AC that are known to date.

## Methods

The study protocol was approved by our Institutional Review Board. The requirement for informed consent was waived in view of the retrospective nature of the study.

### Inclusion and exclusion criteria

One hundred and thirty-two of 351 shoulder MRI scans performed at our institution between January and December 2016 were cases of MR arthrography and excluded, leaving medical records for 219 consecutive patients for retrospective analysis. The inclusion criteria were as follows: restricted passive motion of ≥30 degrees in two or more planes of motion in comparison with the contralateral shoulder; persistent shoulder pain for at least 1 month but no more than 9 months (Hannafin stage 1 or 2) [18]; and no abnormal findings on plain radiographs [9, 12, 19–21]. The reason for our study inclusion criteria was symptoms for ≥1 month to exclude patients with transient symptoms that were not associated with AC and would resolve spontaneously. Seventeen of 46 patients who met these criteria were subsequently excluded because of limited bilateral shoulder ROM, rotator cuff tear, calcified tendinitis, rheumatoid arthritis, and severe osteoarthritis based on MRI findings and clinical assessment. Finally, 29 patients (12 male, 17 female; mean age 51 [range, 30–73] years) were included in the analysis.

### Clinical assessment

An orthopedic surgeon with 24 years of experience performed the physical examinations in all patients before the MRI examination. A universal goniometer was used to assess maximum passive ROM for external rotation, internal rotation, forward flexion, and abduction. External rotation was measured as the maximum angle created by rotating externally with 90 degrees of elbow flexion in the neutral position. Internal rotation was measured as the position of the spinous process reached by the thumb when reaching back with the arm. ROM was quantified by assigning one point for the pelvic region below the fifth vertebrae, two points for the fifth lumbar spinous process, and adding one more point for each segment above [15]. Forward flexion was measured as the maximum arm-trunk angle when the arm was extended forward and elevated as high as possible. Abduction was measured as the maximum arm-trunk angle when the arms were elevated as much as possible to the side.

The severity of shoulder pain was measured using a visual analog scale (VAS) based on a questionnaire administered on the same day as the physical examination. Pain was categorized as pain at rest, pain at night, pain during motion, and worst pain; each patient was instructed to rate the severity of each type of pain as a VAS score.

### MRI acquisition

All patients underwent the same imaging protocol using a 3-T MRI scanner (Intera Achieva, Philips Healthcare, Andover, MA, USA) with a dedicated shoulder coil. During imaging, patients were in the supine position with their arms externally rotated as much as possible. The images were acquired using the following imaging protocol:

- Oblique sagittal fat-suppressed proton density VISTA (volume isotropic turbo spin echo acquisition) sequence with SPAIR (spectral attenuated inversion recovery) imaging (repetition time/echo time [TR/TE], 2000/18.6; echo-train length, 140; section thickness, 1.2 mm; matrix, 268 × 267; field of view [FOV], 160 × 160 mm
- Oblique coronal fat-suppressed T2-weighted imaging (TR/TE, 4700/80; echo-train length, 10; section thickness, 3 mm; matrix, 356 × 255; FOV, 160 × 160 mm)
- Oblique coronal T1-weighted imaging (TR/TE, 530/20; echo-train length, 3; section thickness, 3 mm; matrix, 358 × 258; FOV, 160 × 160 mm)
- Oblique sagittal T2-weighted imaging (TR/TE, 3800/80; echo-train length, 16; section thickness, 3 mm; matrix, 356 × 256; FOV, 160 × 160 mm)
- Oblique sagittal T1-weighted imaging (TR/TE, 530/20; echo-train length, 3; section thickness, 4 mm; matrix, 356 × 258; FOV, 160 × 160 mm)
- Axial fat-suppressed proton density imaging (TR/TE, 2100/30; echo-train length, 20; section thickness, 3 mm; matrix, 356 × 240; FOV, 160 × 160 mm).

### MRI analysis

Assessment and measurements on all MRI images were performed by two musculoskeletal radiologists, each with 9 years of experience and working independently, using a PACS (picture archiving and communication system; INFINITT, Infinitt Healthcare, Seoul, Korea). The radiologists were blinded to all clinical information. Quantitative and qualitative MRI findings for the diagnosis of AC were based on the existing literature [2, 8, 12, 22]. Before the analysis, a training session was conducted for both radiologists using images that were different from those analyzed in the study. Data measured independently by the radiologists were used for assessment of interobserver variance and all parameters were re-evaluated to reach consensus before using the data for statistical analysis.

#### Quantitative analysis

**Fig. 1:**
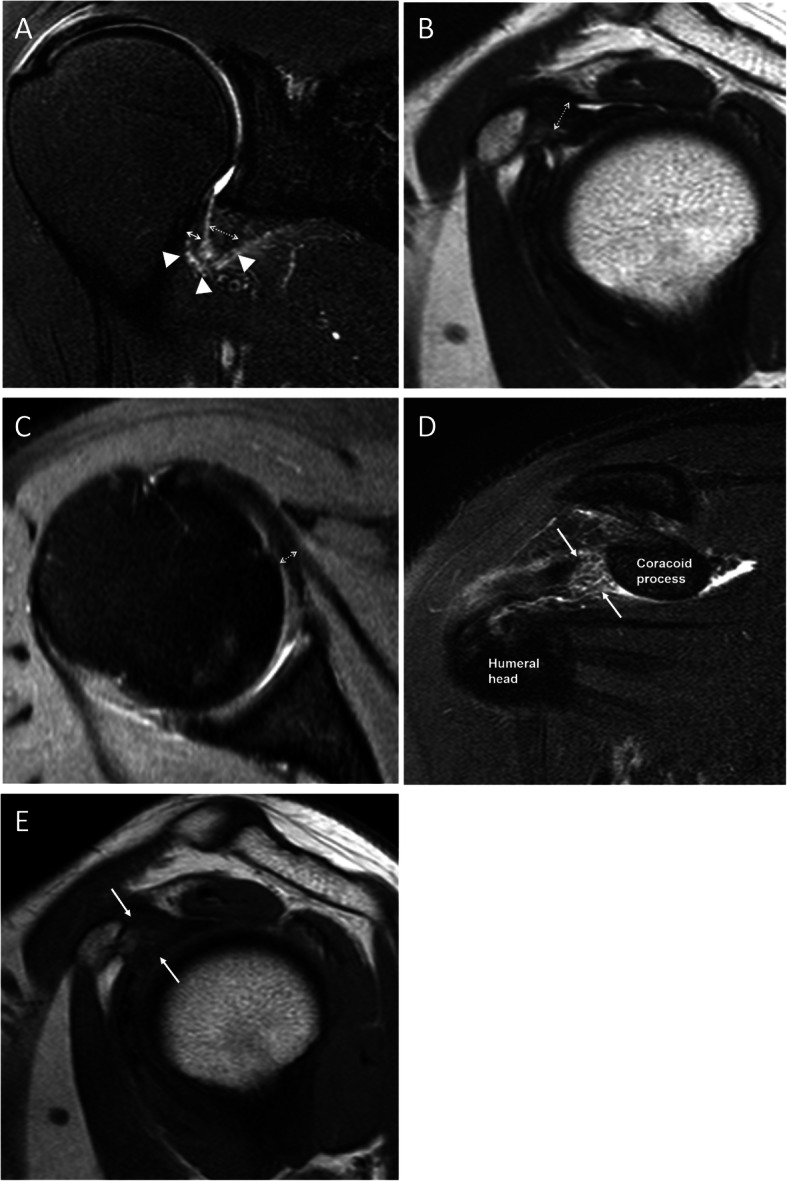
Examples of findings on magnetic resonance images for a 48-year-old woman with adhesive capsulitis. **a**. Oblique coronal fat-suppressed T2-weighted image showing measurement of the thickest portion of the axillary joint capsule in both humeral (arrow) and glenoid (dashed arrow) attachment and also showing axillary capsular thickening and abnormal hyperintensity (arrow heads). Increased thickness was present only at the glenoid portion (6.23 mm); thickness was normal at the humeral portion (2.81 mm). **b** Oblique sagittal T2-weighted image showing measurement of the coracohumeral ligament thickness (dashed arrow). **c** Axial fat-suppressed proton density image showing measurement of anterior capsular thickness (dashed arrow) below the subscapularis tendon. **d** Oblique coronal fat-suppressed T2-weighted image at the coracoid process level showing abnormal hyperintensity in the subcoracoid fat triangle (arrows). **e** Oblique sagittal T1-weighted image showing obliteration of the subcoracoid fat triangle (arrows)

Oblique coronal fat-suppressed T2-weighted imaging was used to measure the humeral and glenoid capsular thicknesses in the axillary recess; the larger of the two measured values was defined as the maximal axillary capsular thickness (Fig. 1a). Oblique sagittal T2-weighted imaging was used to measure the CHL thickness from the thickest part of the entire ligament (Fig. 1b). Axial fat-suppressed proton density imaging was used to measure the anterior capsular thickness, which was measured from the thickest portion of the area showing hypointensity below the subscapularis muscle (Fig. 1c) [12]. All measured values were recorded up to two decimal points.

#### Qualitative analysis

Qualitative MRI findings were evaluated based on the presence or absence of the following: humeral and glenoid capsular hyperintensity in the axillary joint capsule; anterior capsular hyperintensity; hyperintensity at the rotator interval; and obliteration of the subcoracoid fat triangle. Abnormal hyperintensity was determined by the presence of hyperintensity in each joint capsule and rotator interval using oblique coronal fat-suppressed T2-weighted imaging (Fig. 1a, d), with presence of hyperintensity in either the humeral or glenoid capsule determined as abnormal axillary capsular hyperintensity. Obliteration of the subcoracoid fat triangle was defined as hypointensity of fat relative to the subcutaneous fat on oblique sagittal T1-weighted images (Fig. 1e).

### Statistical analysis

The Wilcoxon signed-rank test was used to compare ROM between the affected and unaffected (contralateral) shoulder. Spearman correlation analysis was used to analyze the correlations between MRI findings and clinical features (ROM, pain, and duration of symptoms). For multiple comparisons, the Benjamini-Hochberg procedure for controlling the false discovery rate was used. The Benjamini-Hochberg adjusted *P*-value < 0.05 were considered statistically significant [23, 24].

For assessment of interobserver agreement, the intraclass correlation coefficient (ICC) was calculated for quantitative analysis and Cohen’s kappa was calculated for qualitative analysis. The ICC or kappa value was interpreted as follows: 0 = poor agreement; 0.01–0.20 = slight agreement; 0.21–0.40 = fair agreement; 0.41–0.60 = moderate agreement; 0.61–0.80 = good agreement; and 0.81–1.00 = excellent agreement.

All statistical analyses were performed using SPSS version 20 (IBM Corp., Armonk, NY, USA).

## Results

Table 1 shows the clinical characteristics of the study population, which comprised 12 men and 17 women of mean age 51.2 years. The mean interval between physical examination and MRI was 16 (range, 4–48) days. The mean duration of symptoms was 5 (range, 1–9) months, meaning that only patients with early stage AC were included. The VAS pain score tended to increase in the order of pain at rest, pain at night, pain during motion, and worst pain. Comparison of ROM between the affected and unaffected sides showed a significant decrease in ROM of the affected shoulder in all directions.

**Table 1:** Demographic variables, clinical data, and MRI findings in the study participants (*n* = 29)

Characteristic	Value		
**Demographics**			
Age, years	51.21 ± 9.18 (30–73)		
Male/Female	12/17		
**Clinical data**			
Duration of symptoms, months	5.09 ± 3.17 (1–9)		
VAS pain score			
Resting	2.76 ± 2.33 (0–8)		
Night	4.52 ± 2.92 (0–10)		
Motion	6.52 ± 2.46 (2–10)		
Worst	7.83 ± 1.58 (5–10)		
Range of motion, degrees	Affected shoulder	Unaffected shoulder	*P*-value
External rotation	35.17 ± 21.07 (5–85)	69.14 ± 9.74 (50–85)	< 0.001
Internal rotation	6.31 ± 4.22 (1–13)	12.79 ± 2.06 (8–17)	< 0.001
Forward flexion	137.59 ± 22.94 (95–170)	170.34 ± 10.50 (140–180)	< 0.001
Abduction	132.07 ± 34.45 (20–175)	172.07 ± 7.85 (155–180)	< 0.001
**MRI parameters**			
*Quantitative analysis*			
Maximal axillary capsular thickness	7.04 ± 2.29 (1.98–7.81)		
Humeral capsular thickness	6.04 ± 2.84 (0.94–7.56)		
Glenoid capsular thickness	5.80 ± 1.92 (1.13–7.81)		
Coracohumeral ligament thickness	2.99 ± 0.86 (1.13–5.24)		
Anterior capsular thickness	4.01 ± 1.32 (0.70–7.56)		
*Qualitative analysis*			
Hyperintensity in axillary recess	24 (82.75)		
Humeral capsular hyperintensity	21 (72.41)		
Glenoid capsular hyperintensity	22 (75.86)		
Hyperintensity in the rotator interval	22 (75.86)		
Hyperintensity in the anterior capsule	26 (89.66)		

The data are presented as the mean ± standard deviation (range) or as the number (percentage)

### Correlation between MRI findings and clinical features

**Fig. 2:**
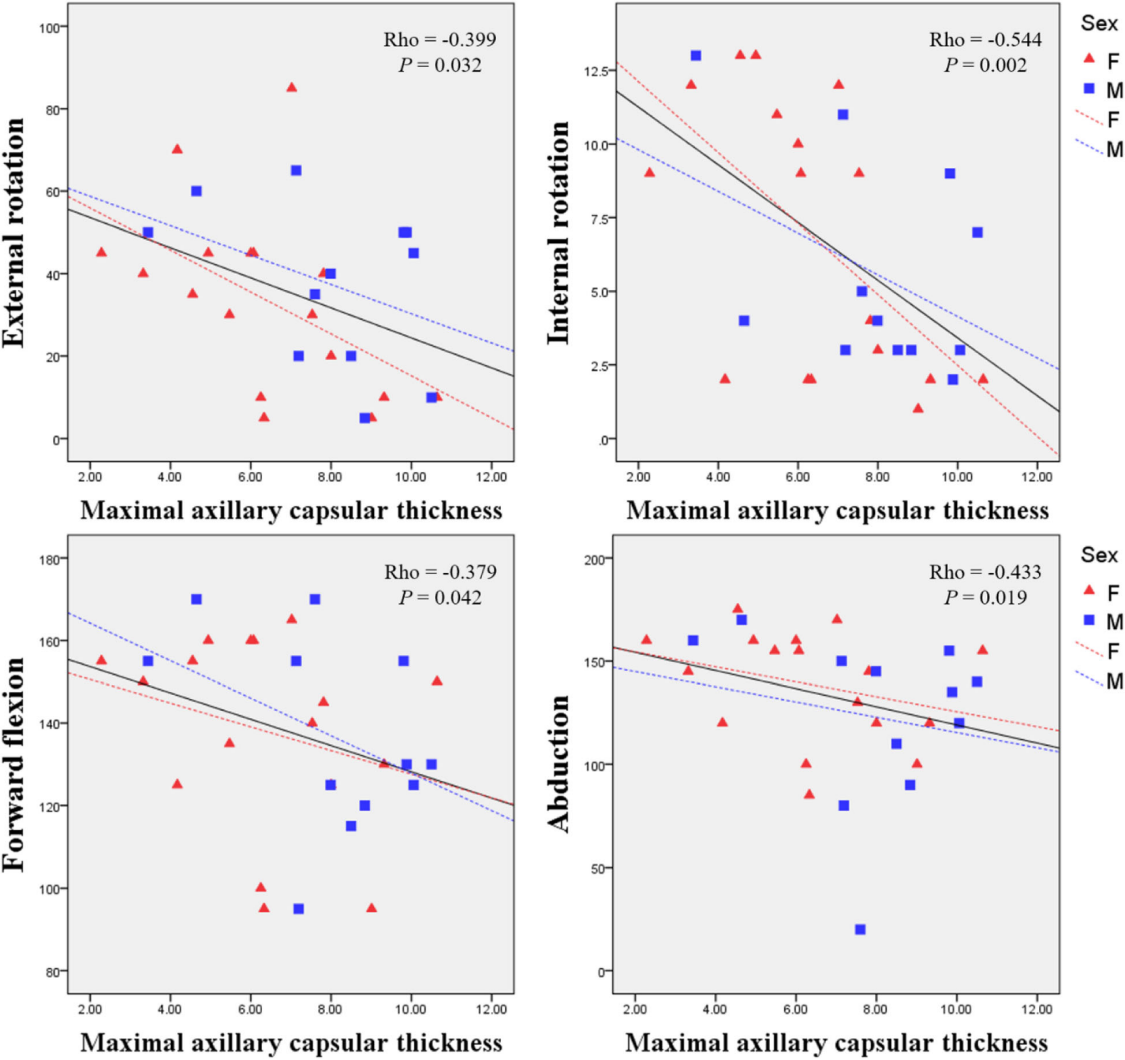
Scattergrams showing the relationships between the four directions of range of motion (ROM) and maximal axillary capsular thickness. Filled squares with dotted lines and filled triangles with dash-dot lines represent the scatter plots with trend lines for men and women, respectively. Bold black lines with statistical values are trend lines for all patients

Table 2 shows the correlation between MRI findings and ROM. In patients with AC, some MRI findings in the axillary recess showed correlations with specific ROM. Maximal axillary capsular thickness (Fig. 2) and humeral thickness were negatively correlated with internal rotation. Also, hyperintensity in axillary recess and glenoid were negatively correlated with abduction. Humeral hyperintensity was negatively correlated with forward flexion. There were no MRI findings that showed correlations with external rotation.

**Table 2:** Correlation analysis between MRI findings and ranges of motion

	External rotation			Internal rotation			Forward flexion			Abduction		
Variable	coefficient (rho)	*P*-value	*B-H* adjusted *P*-value	coefficient (rho)	*P*-value	*B-H* adjusted *P*-value	coefficient (rho)	*P*-value	*B-H* adjusted *P*-value	coefficient (rho)	*P*-value	*B-H* adjusted *P*-value
*Quantitative analysis*												
Maximal axillary capsular thickness	−0.399	0.032	0.117	**−0.544**	**0.002**	**0.029**	− 0.379	0.042	0.123	−0.433	0.019	0.093
Humeral capular thickness	−0.356	0.058	0.150	**−0.564**	**0.001**	**0.022**	−0.393	0.035	0.11	−0.473	0.090	0.180
Glenoid capsular thickness	−0.270	0.157	0.288	−0.423	0.022	0.097	−0.261	0.171	0.289	−0.322	0.088	0.184
Coracohumeral ligament thickness	0.107	0.580	0.709	0.077	0.693	0.726	0.111	0.567	0.713	0.092	0.637	0.738
Anterior capsular thickness	0.080	0.681	0.749	−0.241	0.209	0.307	0.079	0.685	0.735	−0.037	0.849	0.869
*Qualitative analysis*												
Hyperintensity in axillary recess	−0.346	0.066	0.161	−0.418	0.024	0.096	−0.455	0.013	0.082	**−0.532**	**0.003**	**0.033**
Humeral capsular hyperintensity	−0.334	0.077	0.169	−0.396	0.034	0.115	**−0.612**	**0.001**	**0.044**	−0.38	0.042	0.116
Glenoid capsular hyperintensity	−0.480	0.008	0.059	−0.335	0.075	0.174	−0.436	0.018	0.099	**−0.508**	**0.005**	**0.044**
Hyperintensity at the rotator interval	−0.300	0.113	0.216	−0.258	0.177	0.288	−0.082	0.671	0.757	−0.247	0.197	0.299
Hyperintensity in the anterior capsule	−0.254	0.184	0.289	−0.014	0.943	0.943	−0.203	0.291	0.413	−0.141	0.465	0.639
Obliteration of the rotator interval	−0.262	0.170	0.299	0.099	0.611	0.727	−0.114	0.554	0.739	−0.114	0.555	0.718

*B-H* adjusted *P*-value, the Benjamini-Hochberg adjusted *P*-value

In the analysis of the correlation between MRI findings and severity of pain, there were no correlations between MRI findings and severity of pain.

The hyperintensity in the humeral capsule was the only one MRI finding that was correlated with duration of symptoms (rho = − 0.543, adjusted *P-value* = 0.022).

### Interobserver agreement

Table 3 shows a summary of the results for interobserver agreement. All findings for the quantitative analyses showed good agreement (ICC, 0.61–0.71) while those for the qualitative analysis showed moderate-to-good agreement (kappa, 0.43–0.79).

**Table 3:** Interobserver agreement

Parameter	ICC	Kappa	Agreement
*Quantitative analysis*			
Anterior capsular thickness	0.67		Good
Humeral capsular thickness in axillary recess	0.71		Good
Glenoid capsular thickness in axillary recess	0.61		Good
Coracohumeral ligament thickness	0.63		Good
*Qualitative analysis*			
Hyperintensity in the anterior capsule		0.56	Moderate
Humeral capsular hyperintensity in axillary recess		0.79	Good
Glenoid capsular hyperintensity in axillary recess		0.71	Good
Hyperintensity in the rotator interval		0.48	Moderate
Obliteration of the subcoracoid fat triangle		0.43	Moderate

*ICC* Intraclass correlation coefficient

## Discussion

The retrospective study analyzed the correlations between MRI findings and clinical features (ROM, pain, and duration of symptoms) in patients with AC. Axillary capsular thickness and hyperintensity on MRI were negatively correlated with duration of symptoms and ROM in some directions.

Reduced ROM and shoulder pain have a variety of causes, including rotator cuff tear, bursitis, and calcified tendinitis. Therefore, it is important to be able to differentiate between these based on radiological findings [1]. Rotator cuff tear shows hyperintensity on T2-weighted images due to the space created by a partially or completely torn tendon being filled by a watery component, such as joint fluid [25]. Bursitis shows expanded findings due to fluid build-up in the subacromial subdeltoid bursa [26]. Calcified tendinitis shows radiopaque calcified deposits on tendons on plain radiographs and computed tomography scans and T2 hyperintensity is found because of nearby inflammation [27]. If the aforementioned findings are absent on radiologic examination, primary AC with capsular abnormality could be differentiated and diagnosed.

A study by Hannafin et al. [18] used a four-stage classification system based on progression of AC and defined the first stage (0–3 months) and second stage (3–9 months) with reduced ROM as early stages of AC. However, one of our study inclusion criteria was symptoms for ≥1 month to exclude patients with transient symptoms that were not associated with AC and would resolve spontaneously. Although the exact etiology of AC has not been identified, the pathophysiology likely involves capsular inflammation, which subsequently leads to capsular fibrosis [1, 6]. Therefore, AC shows a pattern of pain being dominant in the early stage followed by gradual reduction in ROM due to synovial and capsular inflammation and fibrosis. Capsular fibrosis becomes most severe in the late stage as the inflammation subsides. Although the symptoms of AC are known to improve naturally over time, complete recovery may take up to 2 years [2, 6, 20, 28]. There are various treatments available, including physical therapy, corticosteroid injection, manipulation under anesthesia, and surgical capsular release [13, 18, 29]. The decision regarding such treatment modalities may vary depending on the clinical stage, accurate diagnosis of AC, and accurate identification of AC stage and could ultimately shorten the clinical course [13, 18, 20].

Ahn et al. [15] reported a significant correlation between axillary capsular thickness and ROM in external rotation. The present study also found a negative correlation of axillary capsular thickness with ROM in external rotation and in other directions (internal rotation, forward flexion, and abduction). However, there was an only significant negative correlation between axillary capsular thickness and internal rotation after the adjustment for multiple comparisons, This discordance may be attributed to the fact that the study by Ahn et al. [15] did not differentiate different stages of AC whereas the present study analyzed only patients with early AC. A study by Sofka et al. [17] reported that axillary capsular thickness was significant greater in the early clinical stage (stage 2) than in any other stage but that there were no significant differences between other stages. Therefore, capsular thickening occurred together with capsular inflammation in the early stage of disease, suggesting that such findings may be correlated with clinical features. Given that the present study included only patients with early AC, correlations between capsular thickness in ROM for internal rotation may have appeared, unlike in other studies. Moreover, ROM in internal rotation tended to show a relatively higher correlation than other directions of ROM in the present study. This may have been due to the scoring system used for the degree of internal rotation based on each location in the spine, which may have shown correlations that could not be identified in previous studies [15]. Furthermore, a study by Park et al. [9] reported that hyperintensity in the axillary recess was correlated with ROM in external rotation, and the present study also found correlations between hyperintensity in the axillary recess and ROM in various directions. After the correction of multiple comparisons, there were only significant negative correlations between hyperintensity in axillary recess and abduction/forward flexion. A negative correlation was also found between hyperintensity in the humeral capsule and duration of symptoms, which is determined to be the effect of increased blood flow and edema associated with capsular inflammation in the early stage of AC, similar to results in previous studies [9, 17, 18]. As in previous studies [15, 22], this study also found no correlation between severity of pain and capsular thickness.

According to previous studies, CHL thickening and obliteration of the subcoracoid fat triangle are important diagnostic criteria for AC [2, 30]. While the present study found CHL thickening and obliteration of the subcoracoid fat triangle in most patients, these findings were not correlated with clinical features, as reported previously [9, 15].

This study had some limitations, including the following. First, the number of patients with AC was relatively low, which may have introduced a degree of bias. However, the results are similar to those of previous studies because the study population was limited to patients with early AC and the sample size was large enough to derive statistically valid conclusions. Second, correlations between MRI findings and clinical features at different stages could not be evaluated because no subgroup analysis based on different clinical stages could be performed. Third, patients with AC were defined based on clinical criteria not by diagnosis based on arthroscopy or tissue biopsy. However, because arthroscopic treatment is not recommended as first-line treatment for AC, a histological diagnosis may have been unethical. Therefore, we selected those patients suspected of having AC based on clinical symptoms, in the same way that AC is diagnosed in clinical practice and eliminated the possibility of another disease by MRI findings.

## Conclusions

In conclusion, axillary capsular thickening and hyperintensity on MRI in patients with early AC correlates with limited ROM. MRI findings in patients with AC could be used for indicators of clinical symptoms.

## Data Availability

The datasets generated during and/or analysed during the current study are available from the corresponding author on reasonable request.

## Abbreviations

AC: Adhesive capsulitis
CHL: Coracohumeral ligament
FOV: Field of view
ICC: Intraclass correlation
MRI: Magnetic resonance imaging
ROM: Range of motion
TR/TE: Repetition time/echo time
VAS: Visual analog scale
